# Complete Genome Sequence of Klebsiella aerogenes Siphophage Solomon

**DOI:** 10.1128/MRA.01419-20

**Published:** 2021-01-21

**Authors:** Alexis T. Hudson, James Clark, Jason J. Gill, Mei Liu

**Affiliations:** a Center for Phage Technology, Texas A&M University, College Station, Texas, USA; DOE Joint Genome Institute

## Abstract

Klebsiella aerogenes is a bacterium that can cause a variety of infections. Phage-based biotechnologies may be useful for controlling antibiotic-resistant strains of this bacterium. The characterization of K. aerogenes phage Solomon is described here. Solomon has a 51,775-bp genome, with structural components closely resembling those of Escherichia coli siphophage T1.

## ANNOUNCEMENT

Klebsiella aerogenes (formerly Enterobacter aerogenes) is a Gram-negative bacterium found in feces, water, and the intestinal tract and is capable of causing wound, respiratory, and urinary tract infections (1, 2). Recently, antibiotic-resistant strains have emerged (3). To combat these resistant strains, phage-based approaches could be utilized. Here, the isolation and analysis of K. aerogenes siphophage Solomon are described.

Bacteriophage Solomon was isolated in July 2019 from samples collected from a wastewater treatment plant located in Houston, TX (global positioning system [GPS] coordinates, 29.6433326, −95.2633269) by the soft-agar overlay method (4). It was propagated on K. aerogenes strain ATCC 13048 grown at 37°C on LB broth or agar. The phage was determined to be a siphophage by negative-stain transmission electron microscopy (2% uranyl acetate [wt/vol]) (5) and imaged at the Texas A&M University Microscopy and Imaging Center. The phage DNA was purified using the Promega Wizard DNA extraction system as described previously (6). Libraries were prepared with 300-bp inserts using a Nextera Flex kit and sequenced with paired-end 150-bp reads using V2 300-cycle chemistry on the Illumina MiSeq platform. The quality of the sequence reads (322,670 in total) obtained from the library index containing the phage sample was controlled with FastQC (www.bioinformatics.babraham.ac.uk/projects/fastqc), and the genome sequence was assembled from these reads using SPAdes v3.5.0 (7) into a contig at 89.4× coverage. The genome sequence was closed by PCR and Sanger sequencing of the product amplified by primers 5′-CGAGCAAGCAGGAAACTACA-3′ and 5′-TCCTATCTCCCTGTTATCCGG-3′. The genome was annotated using the tools hosted at the Center for Phage Technology (CPT) Galaxy online at https://cpt.tamu.edu/galaxy-pub via the CPT Galaxy and WebApollo interfaces (8–10) under default settings. GLIMMER v3 (11) and MetaGeneAnnotator v1.0 (12) were used to create the structural annotation, while tRNAs were detected by ARAGORN v2.36 (13). The function of the called genes were predicted by BLAST v2.9.0 (14) against the NCBI nonredundant (nr) and Swiss-Prot databases (15), InterProScan v5.33 (16), and TMHMM v2.0 (17). progressiveMauve v2.4 (18) was used to calculate the genome-wide DNA sequence similarity.

The genome of the siphophage Solomon is 51,775 bp long. It has a GC content of 47.9% and a coding density of 91.3%. There were 82 protein-coding genes predicted, and no tRNA was found. As determined by BLASTn, Solomon is most related to Klebsiella pneumoniae phage Domnhall (GenBank accession no. NC_049835.1; at ∼74% nucleotide identity) and Enterobacter aerogenes phage F20 (NC_043469.1; at ∼88% nucleotide identity), indicating that these phages are circulating among related *Enterobacteriaceae* hosts. Compared with other well-studied phages, the Solomon structural components most closely resemble those of Escherichia coli siphophage T1, with clear protein similarity to the T1 TerL, portal, scaffold, capsid, major tail, and tape measure proteins as determined by BLASTp (E, <0.001). The putative tape measure chaperone and its associated programmed translational frameshift were identified. The lysis cassette includes a putative holin, a SAR endolysin, and a unimolecular spanin. Two potential receptor-binding proteins were identified, namely, a lambda J-like tail spike and a tail fiber with similarity to the E. coli phage T5 L-shaped tail fiber. Solomon encodes its own putative primase and helicase, SSB, and nucleotide kinases for DNA replication. Solomon contains 32 novel genes, with no intron-disrupted genes identified.

### Data availability.

The genome sequence of phage Solomon was deposited under GenBank accession no. MT701592.1 and BioSample accession no. SAMN14609639. The BioProject accession no. is PRJNA222858, and the SRA accession no. is SRR11558350.
